# Supplementary material for: Irradiation-Induced Dysbiosis: The Compounding Effect of High-Fat Diet on Metabolic and Immune Functions in Mice
Source: Int J Mol Sci. 2023 Mar 15;24(6):5631. doi: 10.3390/ijms24065631 (PMC10057711; doi:10.3390/ijms24065631)
Supplement: Supplementary file 1 [file ijms-24-05631-s001.zip › ijms-2253263-supplementary.pdf]

Supplementary Material

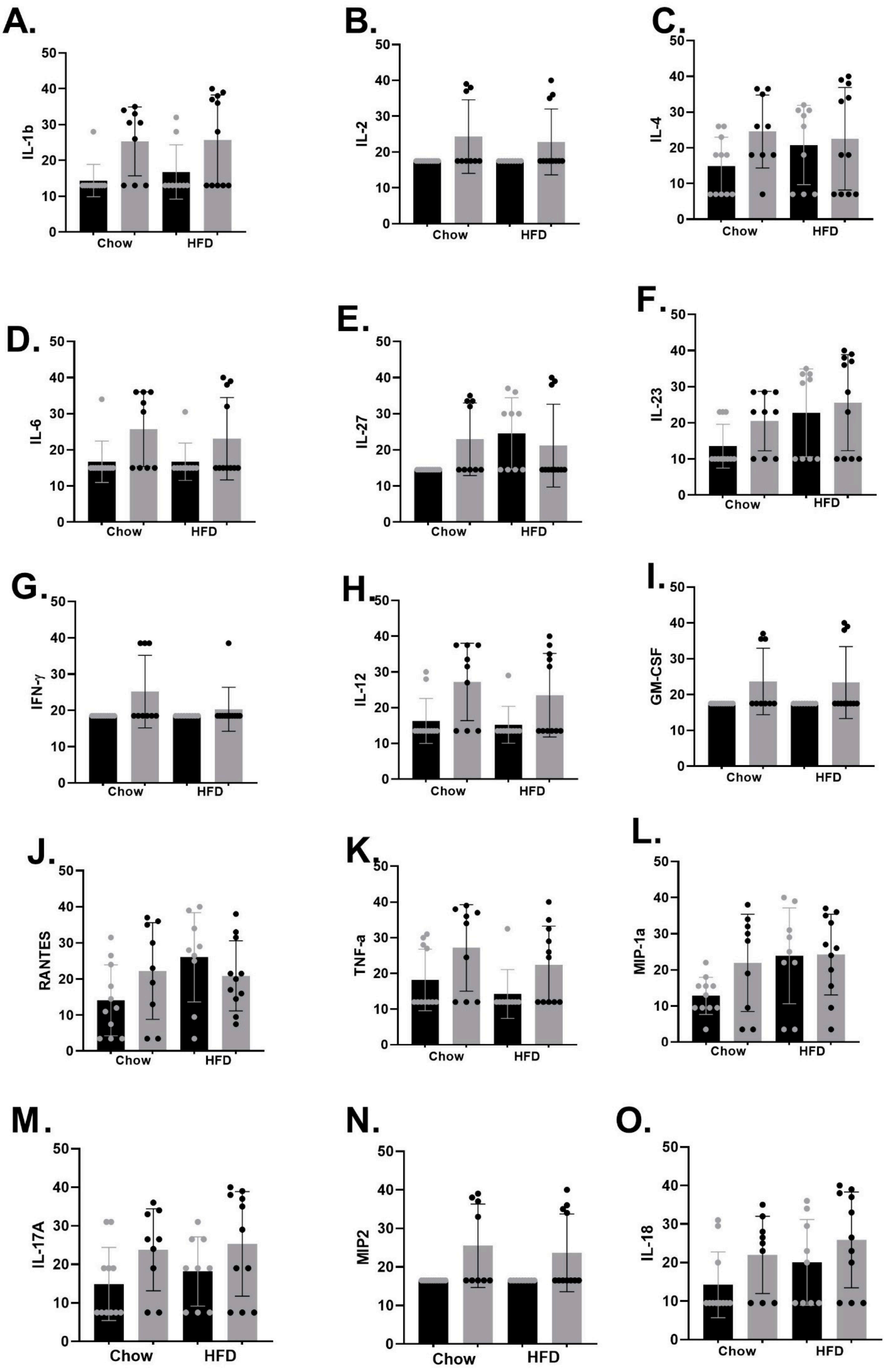

Supplementary Material Figure S1. Inflammatory markers showing no significant between group differences. **A.** IL-1b: interleukin-1β. **B.** IL-2. **C.** IL-4. **D.** IL-6. **E.** IL-27. **F.** IL-23. **G.** IFN-γ: interferon gamma. **H.** IL-12. **I.** GM-CSF: Granulocyte-Macrophage Colony Stimulating Factor. **J.** RANTES: Regulated on Activation Normal T-cell Expressed Secreted/CCL5: C-C Motif Chemokine

Ligand 5. **K.** TNF-a: Tumour Necrosis Factor-α. **L.** MIP-1a: Macrophage Inflammatory Protein-1α/CCL3. **M.** IL-17A. **N.** MIP2: Macrophage Inflammatory Protein-2/CXCL2**.** **O.** IL-18. Black bars and grey dots represent non-irradiated mice. Grey bars and black dots represent irradiated mice.

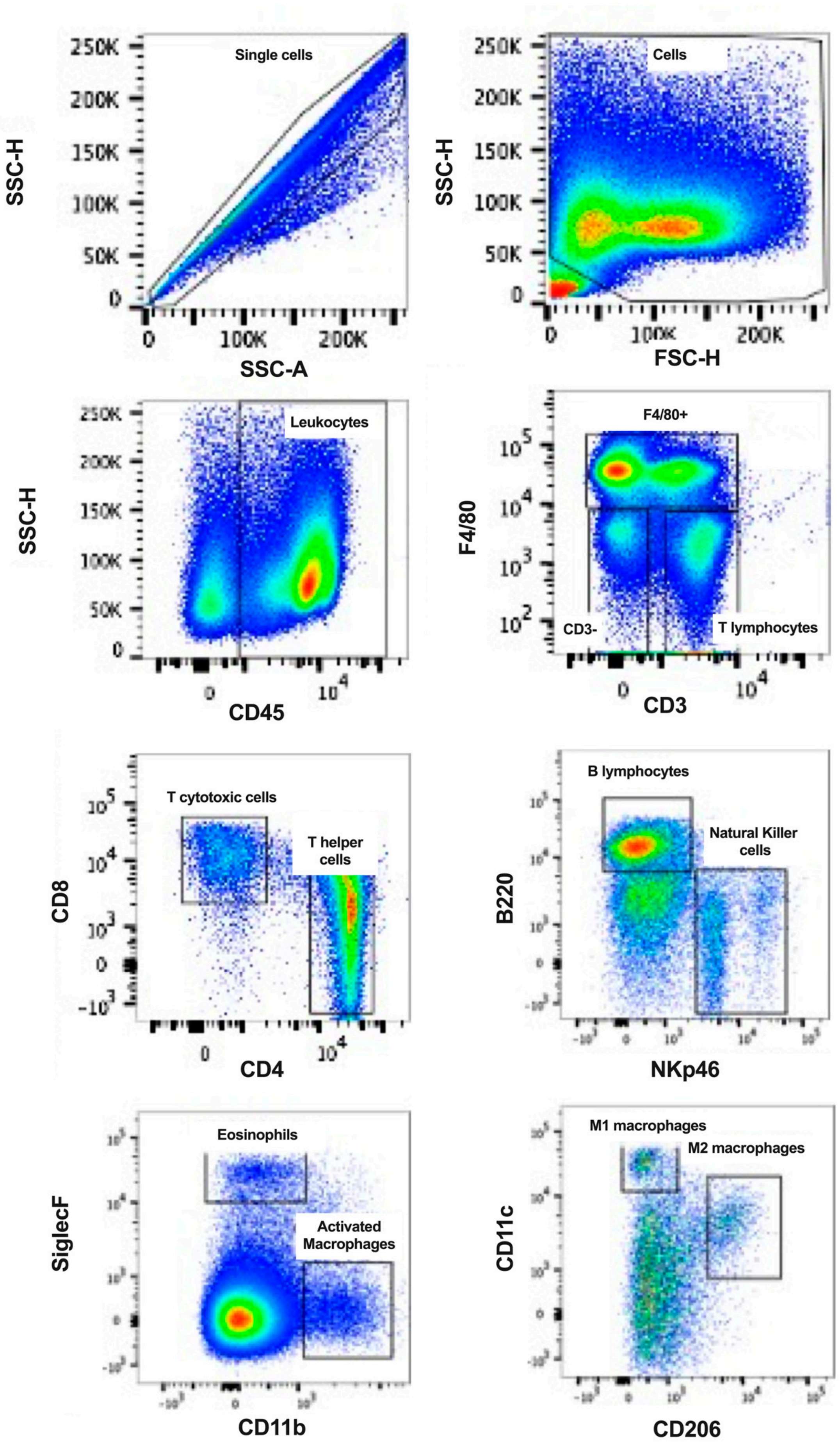
Single cells
SSC-H
SSC-A
Cells
SSC-H
FSC-H
Leukocytes
SSC-H
CD45
F4/80+
F4/80
CD3-
T lymphocytes
CD3
T cytotoxic cells
T helper cells
CD8
CD4
B lymphocytes
Natural Killer cells
B220
NKp46
Eosinophils
Activated Macrophages
SiglecF
CD11b
M1 macrophages
M2 macrophages
CD11c
CD206

Supplementary Material Figure S2. Gating strategy for immune cell profiling in epididymal fat tissue. **A.** Single cells were identified by plotting side scatter area against side scatter height. **B.** Cells were gated on forward scatter vs side scatter. **C.** Leukocytes cells were gated as CD45+ vs side scatter. **D.** T lymphocytes were gated as CD3+ and F4/80- and macrophages were gated as F4/80+. **E.** T lymphocytes were further divided between T helper cells (CD3+CD4+CD8-) and T cytotoxic cells (CD3+CD4-CD8+) by plotting CD4 against CD8. **F.** B lymphocytes (CD3-B220+NKp46-) and NK cells (CD3-B220-NKp46+) were obtained from the CD3- gate, and were distinguished by plotting B220 against NKp46. **G.** Activated macrophages (CD45+F4/80+CD11b+) and eosinophils were obtained from the F4/80+ gate and further divided by plotting CD11b against SiglecF. **H.** M1 (F4/80+CD11b+CD11c+) and M2 (F4/80+CD11b+CD206+) macrophages were gated from the CD11b+ cells and further distinguished by plotting CD11c against CD206.
